# Complications and mortality of cardiovascular emergency admissions during COVID-19 associated restrictive measures

**DOI:** 10.1371/journal.pone.0239801

**Published:** 2020-09-24

**Authors:** Heiko Bugger, Johannes Gollmer, Gudrun Pregartner, Gerit Wünsch, Andrea Berghold, Andreas Zirlik, Dirk von Lewinski

**Affiliations:** 1 Division of Cardiology, Medical University of Graz, Graz, Austria; 2 Institute for Medical Informatics, Statistics and Documentation, Medical University of Graz, Graz, Austria; Medizinische Hochschule Hannover, GERMANY

## Abstract

While hospital admissions for myocardial infarction (MI) and pulmonary embolism (PE) are decreased during the COVID-19 pandemic, controversy remains about respective complication and mortality rates. This study evaluated admission rates, complications, and intrahospital mortality for selected life-threatening cardiovascular emergencies (MI, PE, and acute aortic dissection (AAD)) during COVID-19-associated restrictive social measures (RM) in Styria, Austria. By screening a patient information system for International Statistical Classification of Diseases and Related Health Problems (ICD) diagnosis codes covering more than 85% of acute hospital admissions in the state of Styria (~1.24 million inhabitants), we retrospectively identified patients with admission diagnoses for MI (I21, I22), PE (I26), and AAD (I71). Rates of complications such as cardiogenic shock and cardiopulmonary resuscitation, treatment escalations (thrombolysis for PE), and mortality were analyzed by patient chart review during 6 weeks following onset of COVID-19 associated RM, and during respective time frames in the years 2016 to 2019. 1,668 patients were included. Cumulative admissions for MI, PE and AAD decreased (RR 0.77; p<0.001) during RM compared to previous years. In contrast, intrahospital mortality increased by 65% (RR 1.65; p = 0.041), mainly driven by mortality following MI (RR 1.80; p = 0.042). PE patients received more frequently thrombolysis treatment (RR 3.63; p = 0.006), while rates of cardiogenic shock and cardiopulmonary resuscitation remained unchanged. Of 226 patients hospitalized during RM, 81 patients with suspected COVID-19 disease were screened for SARS-CoV-2 infection with only 5 testing positive. Thus, cumulative hospital admissions for cardiovascular emergencies decreased during COVID-19 associated RM while intrahospital mortality increased.

## Introduction

Coronavirus disease (COVID-19) is an infectious disease caused by acute respiratory syndrome coronavirus-2 (SARS-CoV-2). According to the world health organization, the current COVID-19 pandemic sums up to more than 24 million confirmed infections worldwide, and more than 800,000 deaths have been attributed to COVID-19 (www.covid19.who.int). Pre-existing cardiovascular disease (CVD) seems to increase susceptibility for COVID-19 disease, and 8–12% of patients with COVID-19 have been reported to develop cardiac injury, increasing to about 20% when patients are referred to intensive care units [1–4]. In addition, cases of COVID-19 associated myocarditis and stress cardiomyopathy, and increased mortality of patients with impaired LV- and RV-function with COVID-19 have been reported [5–7]. Furthermore, increased detection and worse outcome of PE in patients with proven or suspected COVID-19 disease have been reported [8, 9]. Thus, COVID-19 may directly impact cardiovascular disease and outcomes, and a bi-directional association between COVID-19 and CVD may exist where one increases and worsens the prognosis of the other [2, 10].

Beyond direct effects of COVID-19, several studies have shown that pandemic-induced but virus-independent effects lead to reduced hospital admissions for acute coronary syndrome (ACS), MI or PE, compared to admission rates before onset of the pandemic or compared to previous years [9, 11–13]. Controversy exists however, whether this decrease in admissions during the pandemic is associated with a parallel increase in mortality or complications, or whether these remain unaffected [14–17]. Given the significant incidence of acute coronary events and PE in the general population, a thorough evaluation of the frequency and potential causes of complications and mortality of cardiovascular emergencies is imperative to assess overall virus-dependent and–independent impact on cardiovascular emergencies during the COVID-19 associated pandemic. In the current study, we performed a real-world analysis comparing admission rates, complications and mortality of selected pre-defined life-threatening cardiovascular emergencies (MI, PE, AAD) during and outside of strict COVID-19 associated RM by interrogating a patient information system covering more than 85% of acute hospital admissions in the state of Styria, Austria, comprising a general population of about 1.24 million people.

## Materials and methods

### Study design

In Styria, a patient information system is installed to optimize communication and information transfer of health data among hospitals. It manages health data of 11 hospitals and hospital alliances, thereby covering more than 85% of acute hospital admissions and 100% of cardiac interventions in Styria, harboring a total population of about 1.24 million people (www.statistik.at). Using this database, we performed a retrospective analysis of pre-defined ICD-10 diagnosis codes indicating hospital admission for MI (I21, I22), PE (I26), and AAD (I71) during the six weeks following onset of mandatory COVID-19 associated RM in Styria (March 16^th^ to April 26^th^ 2020), during the six weeks before onset of RM (February 3^rd^ to March 15^th^ 2020), and during equivalent time frames (6 calendar weeks) in the years 2016 (March 21^st^ to May 1^st^), 2017 (March 20^th^ to April 30^th^), 2018 (March 19^th^ to April 29^th^), and 2019 (March 18^th^ to April 28^th^). RM in Austria were strict, including to stay home, unless grocery shopping or medical service is required, and not to gather with anyone outside the family. RM were maintained during the entire study period. Patients matching any of these ICD-10 codes were individually analyzed by patient chart review to confirm that the day of admission matched the exploration period, that the ICD-10 code matched the diagnosis, that the ICD-10-encoded diagnosis was the primary reason for hospital admission, and to extract information on complications and mortality. The research ethics board of the Medical University of Graz provided ethics approval for the study (32–413 ex 19/20). All patient data were anonymized before accessing the database, and the ethics committee waived the requirement for informed consent. Patient records were accessed between May 11th and May 19th. All the authors vouch for the completeness and accuracy of the data and the analyses.

### Definitions

Inclusion of patients matching I21 was restricted to myocardial infarctions related to an acute atherosclerotic cause. MI was diagnosed and classified (STEMI, NSTEMI) according to the most recent ESC guidelines on diagnosis and treatment of STEMI and NSTE-ACS [18, 19]. In order not to miss any MI that were encoded with a different ICD-10 code than I21, I22 was included in the screening reflecting subsequent STEMI and NSTEMI. To evaluate clinical outcomes of MI patients, we analyzed whether patients (1) suffered cardiogenic shock before, during or shortly after cardiac intervention, or during non-invasive treatment, (2) required CPR before or during cardiac intervention, or during conservative treatment, or (3) died following admission to the hospital and death was attributed to consequences of MI. PE patients were identified by screening for ICD-10 code I26. Definitive diagnosis of PE required conformation by computerized tomography (CT) pulmonary angiogram, ventilation/perfusion scan, or autopsy. To evaluate clinical outcomes of PE, we analyzed whether patients (1) had signs of right heart dysfunction and increased right ventricular pressure using echocardiography or CT, or (2) required thrombolysis, or (3) died following admission to the hospital and death was attributed to consequences of PE. AAD patients were identified by screening for ICD-10 code I71, and definitive diagnosis of AAD required conformation by CT angiography. The following exclusion criteria were predefined: Incorrect ICD code; admission outside the exploration period; non-atherosclerotic MI; unstable angina; accidental diagnosis; diagnosis not reason for admission; traumatic dissection; age under 18 years of age.

### Statistical analysis

The characteristics of the patients are reported as mean (standard deviation, SD) and median (interquartile range) for continuous variables, and the number and percentages are given for categorical variables. The study period (= 2020 during restrictive measures; March 16^th^ to April 26^th^ 2020, calendar weeks 12–17) was compared to the same six calendar weeks in the previous four years (2016–2019) or to the six weeks before onset of RM in 2020. The number of cases was compared using Poisson regression. Rate ratios (RR) and 95% confidence intervals are presented for admissions for MI, PE, and AAD, as well as for patient characteristics, complications and mortality. Since each period to be explored consisted of the same amount of days (six weeks, i.e. 42 days), no offset term was included. All statistical analyses were performed with R version 3.6.1, with a two-sided p-value of 0.05 being considered significant. No adjustment for multiple testing was done.

## Results

**Fig 1:**
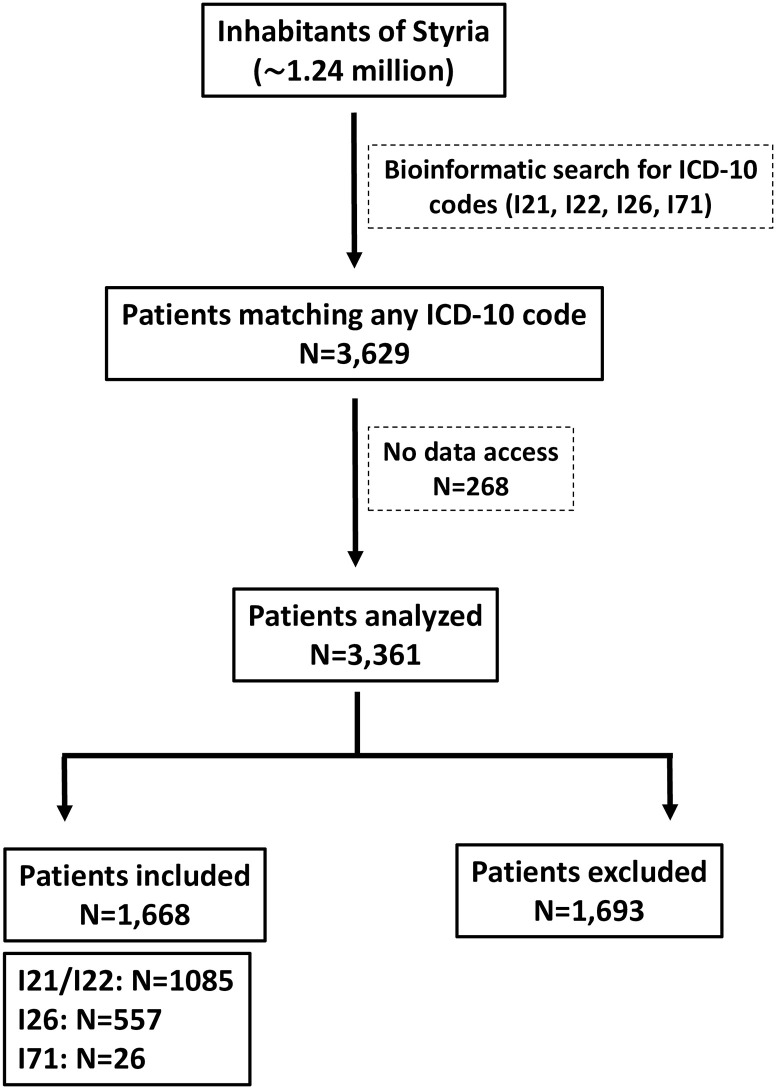
Flow chart of the study and analysis.

Hospital admissions for MI, PE, and AAD that occurred during the first six weeks following onset of COVID-19 associated RM in Styria were quantified, analyzed, and compared to admissions that occurred during respective time frames in the years 2016, 2017, 2018, and 2019, or to admissions that occurred during the six weeks before onset of RM. As illustrated in Fig 1, screening of the patient information system revealed a total of 3,629 hospital admissions in Styria matching any of the ICD-10 diagnoses I21, I22, I26 or I71. For 268 cases patient data could not be accessed due to data protection constraints. Thus, 3,361 admissions underwent individual analysis for inclusion and exclusion criteria, resulting in a final inclusion of 1,668 patients in this study. Patients were excluded because reason for admission was different from predefined ICD-10 codes of cardiovascular emergencies (816), data were missing (54), admission occurred outside the predefined time periods (706), incorrect diagnosis (26), or cardiovascular event occurred during hospital stay.

Characteristics of the patients admitted during the study period and previous years are displayed in Table 1. The mean (SD) age of all patients across years ranged between 67.1 (16.2) and 70.2 (15.3) years. 58.8% to 64.6% of patients were male and 35.4% to 41.2% of patients were female. Of all MI patients analyzed in this study, 64% were diagnosed an NSTEMI while 36% suffered a STEMI. PE admissions were categorized in central, segmental and subsegmental PE. 34%, 37% and 29% suffered central, segmental or subsegmental PE, respectively. Of the 226 patients included during RM in 2020, a total of 81 patients (35.8%) with suspected COVID-19 disease was tested for SARS-CoV-2 infection, with only 5 of 81 patients (6.2%) showing a positive result.

**Table 1:** Patient characteristics and admission numbers during RM (2020 during RM) and previous years (2016–2019).

	2016 (N = 305)	2017 (N = 267)	2018 (N = 318)	2019 (N = 280)	2020 during RM (N = 226)
**Sex**					
**Male, n (%)**	189 (62.0)	157 (58.8)	191 (60.1)	181 (64.6)	142 (62.8)
**Female, n (%)**	116 (38.0)	110 (41.2)	127 (39.9)	99 (35.4)	84 (37.2)
**Age**					
**Mean (SD)**	70.2 (15.3)	68.2 (14.0)	67.1 (16.2)	68.7 (14.6)	68.6 (13.4)
**Median (Q1, Q3)**	73.0 (61.0, 81.0)	68.0 (59.0, 79.0)	70.0 (57.0, 79.0)	71.0 (59.0, 80.0)	70.0 (59.0, 78.0)
**<75, n (%)**	161 (52.8)	167 (62.5)	195 (61.3)	166 (59.3)	138 (61.1)
**≥75, n (%)**	144 (47.2)	100 (37.5)	123 (38.7)	114 (40.7)	88 (38.9)
**Admission**					
**MI, n (%)**	202 (66.2)	173 (64.8)	195 (61.3)	197 (70.4)	148 (65.5)
**PE, n (%)**	97 (31.8)	92 (34.5)	117 (36.8)	76 (27.1)	75 (33.2)
**AAD, n (%)**	6 (2.0)	2 (0.7)	6 (1.9)	7 (2.5)	3 (1.3)
**MI+PE, n (%)**	299 (98.0)	265 (99.3)	312 (98.1)	273 (97.5)	223 (98.7)
**MI+PE+AAD, n (%)**	305 (100)	267 (100)	318 (100)	280 (100)	226 (100)
**Type of MI**					
**NSTEMI, n (%)**	136 (67.3)	111 (64.2)	127 (65.5)	124 (63.3)	91 (61.5)
**STEMI, n (%)**	66 (32.7)	62 (35.8)	67 (34.5)	72 (36.7)	57 (38.5)
**Type of PE**					
**Segmental, n (%)**	39 (42.9)	27 (29.3)	33 (28.7)	27 (35.5)	17 (23.3)
**Subsegmental, n (%)**	30 (33.0)	37 (40.2)	59 (51.3)	16 (21.1)	27 (37.0)
**Central, n (%)**	22 (24.2)	28 (30.4)	23 (20.0)	33 (43.4)	29 (39.7)
**Type of AAD**					
**A, n (%)**	2 (33.3)	2 (100.0)	4 (66.7)	5 (71.4)	3 (100.0)
**B, n (%)**	4 (66.7)	0 (0.0)	2 (33.3)	2 (28.6)	0 (0.0)

**Fig 2:**
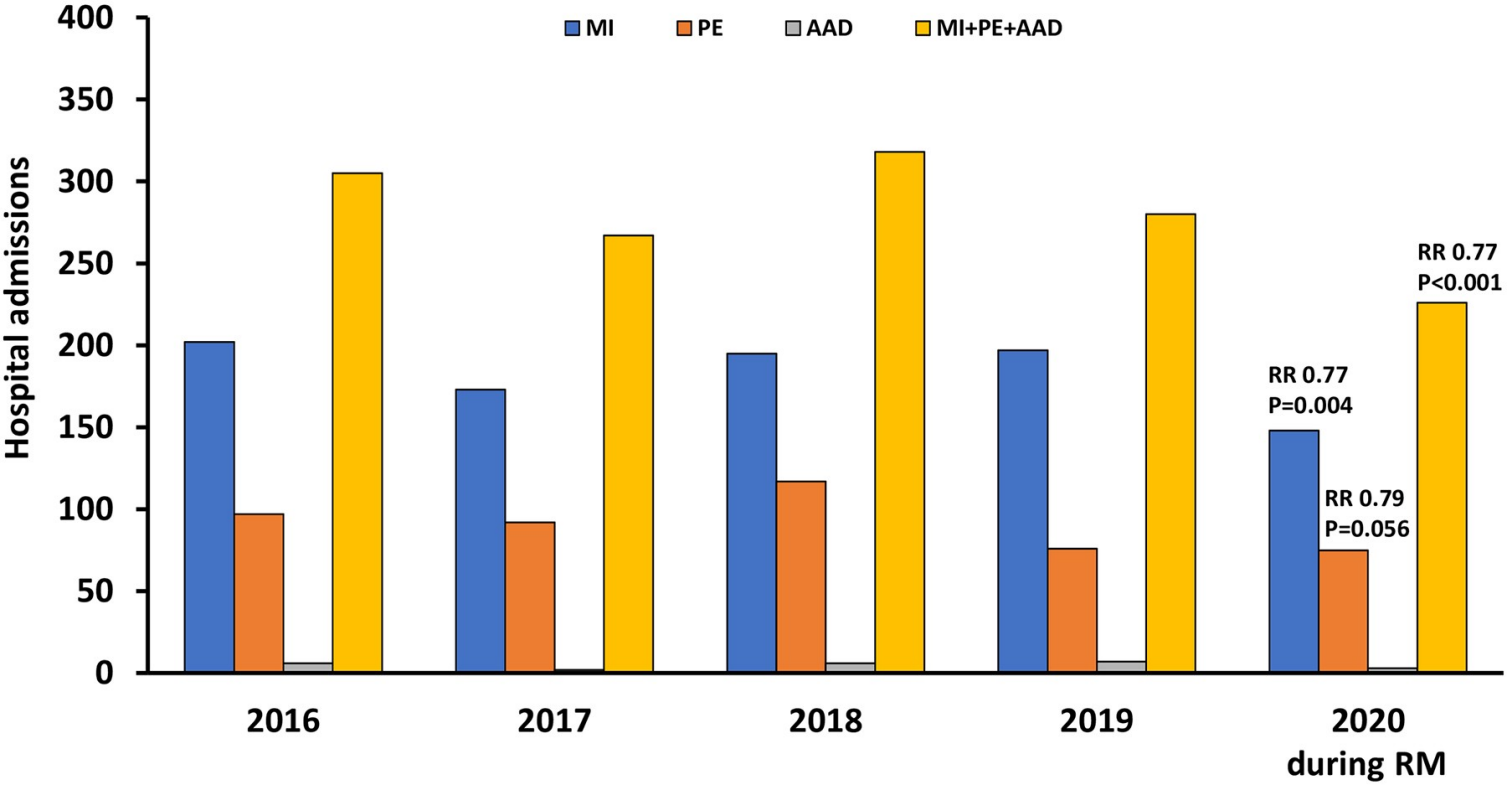
Decreased hospital admissions for cardiovascular emergencies during COVID-19 associated RM. Shown are hospital admissions for MI (blue), PE (orange), AAD (grey), and for cumulative admissions (yellow) during the first six weeks following onset of RM in Styria (= 2020 during RM) and during respective time frames in the years 2016, 2017, 1018 and 2019. Number of cases were compared using Poisson regression, and RR were calculated.

Fig 2 shows the number of hospital admissions for MI, PE, AAD, or the combination of all admission diagnoses. Cumulative admissions were 226 in 2020 during RM, and 280, 318, 267 and 305 during respective episodes in 2019, 2018, 2017 and 2016, respectively (Table 2). Using Poisson regression analysis, the RR for cumulative admissions reflecting the three major life-threatening cardiovascular emergencies was significantly lower during COVID-19 associated RM compared to previous years (RR 0.77, p<0.001). This decrease was driven by lower MI admissions (RR 0.77, p = 0.004) (Table 2). A similar decrease was observed for PE admissions, although significance was not achieved (RR 0.79; p = 0.056). A decrease in cumulative admissions for MI, PE and AAD was also observed when comparing the study period to the six weeks before onset of RM (272 before RM versus 226 in 2020 during RM; RR 0.83, p = 0.040) (S1 and S2 Tables). In each comparison, admission numbers for AAD were low and not different across years (Table 2, S3 Table). No difference was observed for any admission diagnoses across years when performing subgroup analyses for patients aged 75 years and above, or younger than 75 years (S1 Table).

**Table 2:** Complications and mortality of admissions during COVID-19 associated RM (2020 during RM) and previous years (2016–2019).

	2016	2017	2018	2019	2020 during RM	RR (95% CI)	P value
**MI, n**	202	173	195	197	148	0.77 (0.64, 0.92)	0.004
**Cardiogenic shock, n (%)**	19 (9.4)	15 (8.7)	21 (10.8)	15 (7.7)	19 (12.8)	1.40 (0.82, 2.28)	0.191
**CPR, n (%)**	15 (7.4)	8 (4.6)	12 (6.2)	11 (5.6)	11 (7.4)	1.23 (0.61, 2.29)	0.530
**Death, n (%)**	10 (5.0)	11 (6.4)	14 (7.2)	11 (5.6)	16 (10.8)	1.80 (0.99, 3.11)	0.042
**MI (STEMI), n**	66	62	67	72	57	0.85 (0.64, 1.13)	0.279
**Cardiogenic shock, n (%)**	16 (24.2)	11 (17.7)	15 (22.4)	13 (18.1)	16 (28.1)	1.36 (0.76, 2.32)	0.276
**CPR, n (%)**	13 (19.7)	7 (11.3)	10 (14.9)	11 (15.3)	10 (17.5)	1.14 (0.54, 2.19)	0.706
**Death, n (%)**	8 (12.1)	9 (14.5)	9 (13.4)	9 (12.5)	11 (19.3)	1.47 (0.71, 2.80)	0.263
**MI (NSTEMI), n**	136	111	127	124	91	0.73 (0.58, 0.91)	0.006
**Cardiogenic shock, n (%)**	3 (2.2)	4 (3.6)	6 (4.7)	2 (1.6)	3 (3.3)	1.09 (0.25, 3.32)	0.886
**CPR, n (%)**	2 (1.5)	1 (0.9)	2 (1.6)	0	1 (1.1)	1.09 (0.06, 6.77)	0.936
**Death, n (%)**	2 (1.5)	2 (1.8)	5 (3.9)	2 (1.6)	5 (5.5)	2.49 (0.78, 6.84)	0.091
**PE, n**	97	92	117	76	75	0.79 (0.61, 1.00)	0.056
**RV dysfunction, n (%)**	16 (17.8)	17 (18.7)	25 (21.9)	18 (24.0)	18 (24.0)	1.17 (0.68, 1.91)	0.553
**Thrombolysis, n (%)**	2 (2.2)	4 (4.3)	3 (2.6)	2 (2.6)	8 (10.7)	3.63 (1.40, 8.96)	0.006
**Death, n (%)**	1 (1.0)	4 (4.3)	8 (6.8s)	4 (5.3)	5 (6.7)	1.50 (0.49, 3.79)	0.427
**AAD, n**	6	2	6	7	3	0.57 (0.13, 1.66)	0.365
**Death, n (%)**	2 (33.3)	1 (50.0)	1 (16.7)	2 (28.6)	1 (33.3)	1.17 (0.06, 6.83)	0.887
**MI+PE, n**	299	265	312	273	223	0.78 (0.67, 0.89)	0.001
**Death, n (%)**	11 (3.7)	15 (5.7)	22 (7.1)	15 (5.5)	21 (9.4)	1.72 (1.02, 2.76)	0.032
**MI+PE+AAD, n**	305	267	318	280	226	0.77 (0.67, 0.89)	<0.001
**Death, n (%)**	13 (4.3)	16 (6.0)	23 (7.2)	17 (6.1)	22 (9.7)	1.65 (1.00, 2.62)	0.041

Poisson regression: 2020 (during RM) compared to previous years; RM, restrictive social measures.

**Fig 3:**
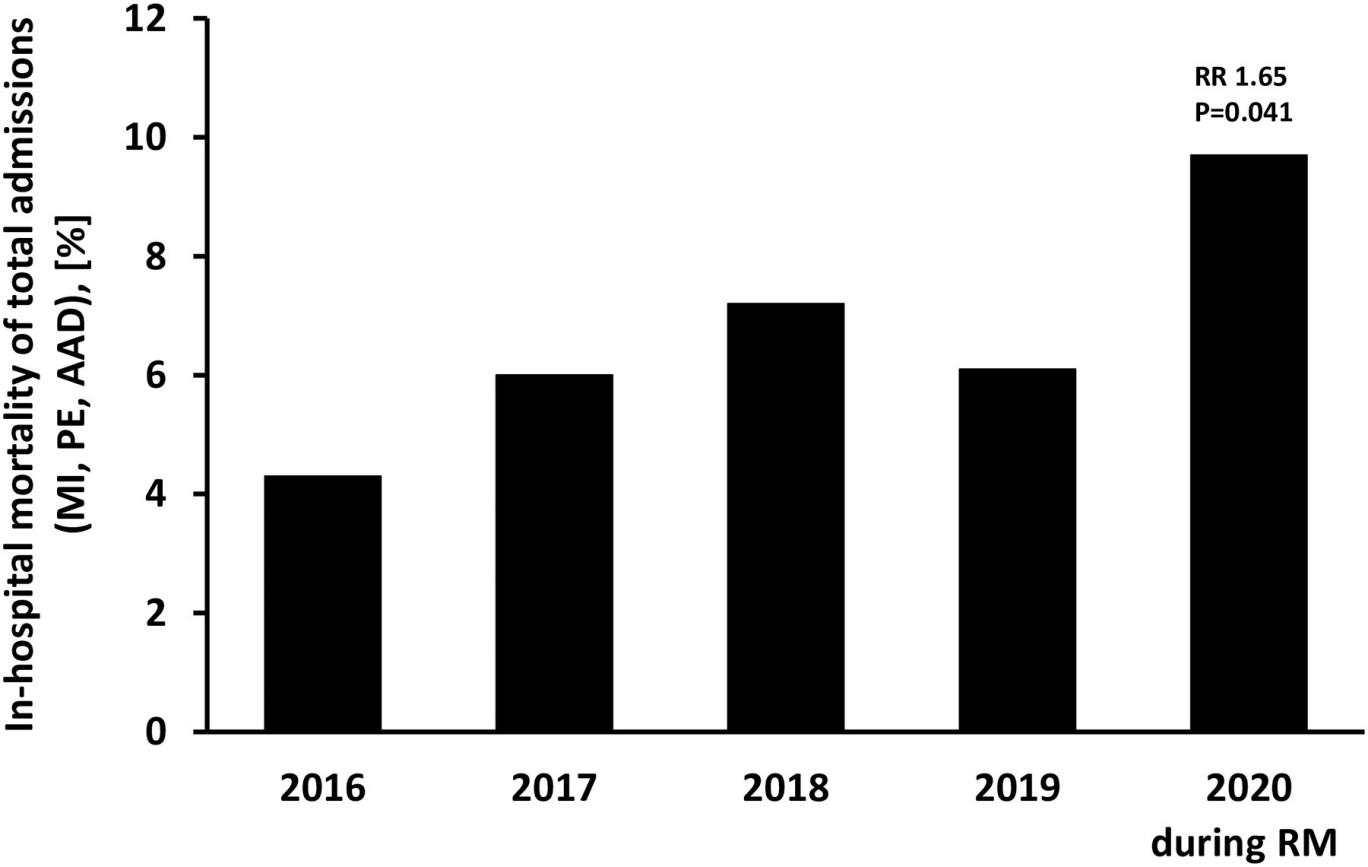
Increased intrahospital mortality of patients admitted for cardiovascular emergencies during COVID-19 associated RM. Shown is intrahospital mortality of cumulative admissions for MI, PE or AAD during the first six weeks following onset of RM in Styria (= 2020 during RM) and during respective time frames in the years 2016, 2017, 2018 and 2019. Data are presented as percent mortality of cumulative admissions (MI, PE, AAD). Comparisons were analyzed using Poisson regression, and RR were calculated.

Complications and mortality of cardiovascular emergencies were analyzed. Intrahospital mortality of cumulative admissions for MI, PE and AAD was 9.7% and thus increased by 65% during COVID-19 associated RM compared to previous years (RR 1.65, p<0.041) (Fig 3). This increase in mortality was driven by increased MI mortality (RR 1.80, p = 0.042), whereas mortality was not increased for PE (Table 2). Cardiogenic shock and CPR occurred in similar number of cases across years (Table 2). While cardiogenic shock and CPR appeared more frequent in STEMI than in NSTEMI patients, no significant difference was observed across years within the two subtypes of MI. Patients admitted for PE more often received thrombolytic treatment during COVID-19 associated RM compared to previous years (RR 3.63, p = 0.006), although RV dysfunction was observed at similar rates across years. When comparing the study period to the six weeks before onset of RM, a similar trend towards increased mortality for cumulative admissions was observed (1.39, p = 0.289) although significance was not achieved (S3 Table).

## Discussion

Given that time from symptoms to first medical contact was longer during the COVID-19 pandemic in other studies [20], we speculate that the increase of intrahospital mortality in our study despite fewer admissions during COVID-19 associated RM may result from a delay in seeking medical assistance. This may have prevented timely therapeutic intervention and ultimately increased mortality. The increase in mortality is likely not explained by direct cardiac effects of COVID-19 disease, since 1) only 6.2% of patients admitted for cardiovascular emergencies and tested for COVID-19 had a concomitant SARS-CoV-2 infection, 2) the death of only one of five SARS-CoV-2 positive patients does not explain an increase of 65% in mortality, and 3) the increase in mortality was driven by MI patients, and none of the patients with MI-related deaths tested for SARS-CoV-2 was positive for SARS-CoV-2. In addition to increased intrahospital mortality of cardiovascular emergency admissions in our study, a 58% increase of out-of-hospital cardiac arrests with a concomitant rise of out-of-hospital deaths was reported during the COVID-19 pandemic [21], and increased out-of-hospital unexplained deaths had 3-fold higher diagnoses of pulmonary embolism during the COVID-19 pandemic, suggesting that overall numbers of complications and mortality due to cardiovascular emergencies are even higher. The rather low percentage of COVID-19 infections in the general population may again argue against COVID-19 as the sole trigger of increased mortality. Of note, while impaired accessibility of hospitals and limited system capacity has been discussed to contribute to increased mortality [22], only 48% of intensive care unit capacities and 33% of regular patient care capacities solely dedicated to the treatment of COVID-19 patients were occupied during peak admission times of SARS-CoV-2 positive patients in Styria, implying that the increased mortality of cardiovascular emergency admissions in our study cannot be explained by constraints of the health care system in Styria. Instead, secondary effects that are not directly related to COVID-19 disease such as misinterpretation of symptoms (dyspnea and chest discomfort rather interpreted as sign of respiratory disease by the patient), strict instructions to stay at home, and increased fear of patients to contact SARS-CoV-2 in medical facilities as a consequence of RM and of the impact of the pandemic situation on individual decision making may be responsible for delayed contact with health care professionals. Of note, the authors of an analysis of the FITT-STEMI trial reported that pre-clinical logistic structures, including contact-to-ballon times, were preserved during the COVID-19 pandemic in Germany, supporting the concept that delayed admissions may indeed result from hesitation to contact the medical system [23]. Avoiding health care may similarly be the reason for less new diagnoses of cancer in the Netherlands since the onset of the COVID-19 pandemic, thereby likely delaying timely therapeutic intervention and also impairing prognosis [24]. Finally, a 47% drop in registered new-onset cases of atrial fibrillation was observed during the national lockdown in Denmark. In the event of prolonged or subsequent lockdowns, the risk of undiagnosed atrial fibrillation may also result in increased complications such as stroke, potentially translating into poorer outcomes in patients with atrial fibrillation during the COVID-19 pandemic [25]. Thus, to avoid unintended secondary effects of measures aiming at limiting the spread of SARS-CoV-2 on cardiovascular mortality, cardiologists and other health care practitioners should be alerted to develop complementary personalized approaches of patient care (e.g. hotlines for cardiovascular disease) to counteract the increase in cardiovascular morbidity and mortality that partially outweighs COVID-19 imposed lethality.

While reduced admissions for myocardial infarction have been reported by several groups during the COVID-19 pandemic [11–13], Scholz and colleagues reported no change in intrahospital mortality for patients admitted for acute STEMI in Germany [23]. The discrepancy regarding mortality compared to our study may be related to the fact that their study analysis ended end of March 2020. However, while light RM were announced in the mid of March 2020 in Germany, a strict lockdown was not initiated until March 22 and lasted until early May 2020. In contrast, our study investigated the entire period of strict RM in Austria. Thus, the difference in study times and coverage of RM may have resulted in a less pronounced decrease in STEMI admissions in the study by Scholz et al. (12,6% versus 23%) and thus potentially also in no change in mortality if emergency calls may have been less delayed compared to our study. In addition, different study populations were investigated, which included STEMI and intra-hospital MI in the study by Scholz and colleagues, and STEMI and atherosclerotic NSTEMI patients but not intrahospital MI in our study. Furthermore, potential effects of regional differences in STEMI admissions and thus also on mortality cannot be excluded [26].

A recent study suggests that a fairly high number of false negative results for SARS-CoV-2 infection may be obtained in patients with few or no symptoms during the incubation period using currently available tests, and patients included in our study tested negative for SARS-CoV-2 were usually only tested at admission with no follow-up tests, thus leaving the possibility that an unknown but substantial number of patients may have had undetected SARS-CoV-2 infection [27]. Since increased mortality was driven by MI patients, and none of the tested MI patients that survived or died were SARS-CoV-2 positive, it is rather unlikely that a significant amount of patients may have been SARS-CoV-2 positive. Nevertheless, it remains to be discussed that viral respiratory infections have been proven to increase the risk of ACS as shown for influenza, implying also a potential direct effect of SARS-CoV-2 infections on ACS incidence and outcome during the COVID-19 pandemic [28]. While the incidence of ACS in patients with COVID-19 remains poorly understood, it has been proposed that plaque rupture, coronary spasm or microthrombi owing to systemic inflammation or cytokine storm may trigger ACS in COVID-19 patients [29]. Activated macrophages may secrete collagenases, thereby degrading the fibrous cap of atherosclerotic plaques, which may lead to plaque rupture; activated macrophages may secrete tissue factor and promote thrombus formation when the plaque ruptures; coronary thrombus formation may also be increased by direct endothelial or vascular injury due to SARS-CoV-2 infection [29–32]. Thus, we cannot entirely rule out that undetected SARS-CoV-2 infections may have triggered ACS and potentially also contributed to increased mortality in our study cohort. At least, it remains to be kept in mind that both COVID-19 and secondary effects of the pandemic may impact incidence and/or fatal outcome of MI.

Studies suggest that PE may be a complication of COVID-19. In a retrospective analysis, Bompard et al. detected an overall cumulative incidence of 24% in a total of 135 COVID-19 patients [33]. High rates of coexisting PE were also observed in COVID-19 patients admitted to intensive care units, detected after a median stay of 6 days following ICU admission [34, 35]. In contrast, only 7 of 119 patients (5.9%) suffering from H1N1 pneumonia experienced thrombotic vascular events, with only a single case of PE [36], suggesting a much higher association of PE with COVID-19 compared to other viral pneumonias. In addition to this direct impact of COVID-19 on PE incidence, our study results suggest that also secondary effects of the COVID-19 pandemic may impact treatment and severity of PE. While we observed a trend towards reduced hospital admissions for PE (RR 0.78; p = 0.056), the number of systemic thrombolytic treatments for PE was strikingly higher (RR 3.63, p = 0.006), implying more severe courses of PE admissions. Although not all patients admitted for PE were tested for SARS-CoV-2, the fact that 32 of 75 patients with PE were tested for SARS-CoV-2 and only 2 of these were SARS-CoV-2 positive, suggests that the implied increase in PE severity may not be due to an association with COVID-19, but instead may result from delayed seeking of medical assistance and may be related to pandemic-associated secondary factors as discussed above. In fact, dedicated emergency rooms were implemented in most Styrian hospitals during the pandemic, and diagnostic algorithms recommended early CT scans of the thorax and D-dimer testing, suggesting that the trend towards less PE admissions may even be underestimated since more cases of PE may have been detected compared to times before COVID-19. In addition, Bram et al. described a 2.5-fold reduction in pediatric bone fractures during the COVID-19 pandemic, indicating a profound reduction in physical activity during the pandemic, which may increase the risk for thromboembolic events [37]. Thus, it may be speculated that during RM an even larger discrepancy may exist between potentially increased PE incidence and decreased use of medical care. It appears that aged, less physically active and comorbid individuals that are considered at increased risk for a severe course of COVID-19 may be at increased risk for PE and/or related complications both due to SARS-CoV-2 infection itself and also due to secondary pandemic-associated factors that compromise the use of medical care such as RM or fear of coronavirus infection.

A number of limitations have to be considered. About half of all patients whose death was attributed to MI were not tested for SARS-CoV-2 infection, and a recent study suggests that a fairly high number of false negative results may be obtained in patients with few or no symptoms during the incubation period [27]. Thus, we cannot entirely exclude a direct contribution of COVID-19 to the increase in mortality. However, mortality was driven by MI patients, and none of the tested MI patients that survived or died were SARS-CoV-2 positive. We also cannot reveal why the observed increase in thrombolytic treatments was not accompanied by an increase in right heart dysfunction, since our retrospective analysis did not enable us to reliably analyze individual decision making by the treating physicians. Furthermore, 268 patients were excluded before inclusion or exclusion for data protection reasons, and 54 patients were excluded due to incomplete information, thus we cannot exclude a confounding bias due to these exclusions. Finally, typical limitations of retrospective analyses may potentially apply, including any selection or misclassification bias, unknown confounders, and the hypothesis-generating character of the study.

## Supporting information

S1 Table Patient characteristics for each admission diagnosis and combinations thereof during COVID-19 associated RM (2020 during RM) and previous years (2016–2019). (DOCX)

S2 Table Patient characteristics of admissions during (2020 during RM) and before (2020 before RM) COVID-19 associated RM. (DOCX)

S3 Table Number and mortality of admissions during (2020 during RM) and before (2020 before RM) COVID-19 associated RM. (DOCX)
