# Supplementary material for: Complications and mortality of cardiovascular emergency admissions during COVID-19 associated restrictive measures
Source: PLoS One. 2020 Sep 24;15(9):e0239801. doi: 10.1371/journal.pone.0239801 (PMC7514100; doi:10.1371/journal.pone.0239801)
Supplement: S1 Table — (DOCX) [file pone.0239801.s001.docx]

**S1 Table: Patient characteristics for each admission diagnosis and combinations thereof during COVID-19 associated RM (2020 during RM) and previous years (2016-2019).**

|  | 2016 | 2017 | 2018 | 2019 | 2020 (during RM) | RR (95% CI) | P value |
| --- | --- | --- | --- | --- | --- | --- | --- |
| MI, n | 202 | 173 | 195 | 197 | 170 |  |  |
| Mean Age (SD) | 71.4 (13.4) | 68.9 (13.2) | 69.8 (13.9) | 69.4 (13.8) | 70.2 (13.7) |  |  |
| Median Age  (Q1, Q3) | 73.0 (63.0, 81.0) | 69.0 (59.0, 80.0) | 72.0 (60.0, 79.0) | 70.0 (59.0, 80.0) | 71.5 (60.0, 80.0) |  |  |
| <75 years, n (%) | 105 (52.0) | 110 (63.6) | 116 (59.5) | 118 (59.9) | 98 (57.6) | 1.00 (0.79, 1.26) | 0.972 |
| ≥75 years, n (%) | 97 (48.0) | 63 (36.4) | 79 (40.5) | 79 (40.1) | 72 (42.4) | 0.99 (0.75, 1.30) | 0.966 |
| Male, n (%) | 136 (67.3) | 116 (67.1) | 132 (67.7) | 137 (69.5) | 115 (67.6) | 0.94 (0.75, 1.16) | 0.549 |
| Female, n (%) | 66 (32.7) | 57 (32.9) | 63 (32.3) | 60 (30.5) | 55 (32.4) | 1.14 (0.84, 1.51) | 0.391 |
| PE, n | 97 | 92 | 117 | 76 | 100 |  |  |
| Mean Age (SD) | 67.6 (18.7) | 66.9 (15.3) | 62.8 (18.7) | 67.5 (16.5) | 67.9 (15.5) |  |  |
| Median Age  (Q1, Q3) | 74.0 (55.0, 81.0) | 67.0 (56.2, 79.0) | 68.0 (50.0, 78.0) | 71.0 (59.0, 80.0) | 71.0 (58.0, 79.0) |  |  |
| <75 years, n (%) | 52 (53.6) | 56 (60.9) | 74 (63.2) | 42 (55.3) | 60 (60.0) | 1.09 (0.79, 1.48) | 0.582 |
| ≥75 years, n (%) | 45 (46.4) | 36 (39.1) | 43 (36.8) | 34 (44.7) | 40 (40.0) | 0.87 (0.57, 1.29) | 0.505 |
| Male, n (%) | 50 (51.5) | 40 (43.5) | 55 (47.0) | 38 (50.0) | 53 (53.0) | 1.28 (0.92, 1.75) | 0.134 |
| Female, n (%) | 47 (48.5) | 52 (56.5) | 62 (53.0) | 38 (50.0) | 47 (47.0) | 0.74 (0.49, 1.08) | 0.134 |
| AAD, n | 6 | 2 | 6 | 7 | 2 |  |  |
| Mean Age (SD) | 68.7 (13.6) | 74.0 (7.1) | 60.7 (13.4) | 62.3 (10.9) | 51.5 (0.7) |  |  |
| Median Age  (Q1, Q3) | 68.5 (61.5, 77.0) | 74.0 (71.5, 76.5) | 57.5 (52.5, 68.5) | 65.0 (56.5, 68.5) | 51.5 (51.2, 51.8) |  |  |
| <75 years, n (%) | 4 (66.7) | 1 (50.0) | 5 (83.3) | 6 (85.7) | 2 (100.0) | 1.31 (0.31, 3.94) | 0.666 |
| ≥75 years, n (%) | 2 (33.3) | 1 (50.0) | 1 (16.7) | 1 (14.3) | 0 (0.0) | 0.00 | 0.996 |
| Male, n (%) | 3 (50.0) | 1 (50.0) | 4 (66.7) | 6 (85.7) | 2 (100.0) | 1.00 (0.16, 3.58) | 1.000 |
| Female, n (%) | 3 (50.0) | 1 (50.0) | 2 (33.3) | 1 (14.3) | 0 (0.0) | 1.00 (0.05, 5.62) | 1.000 |
| MI+PE, n | 299 | 265 | 312 | 273 | 223 |  |  |
| Mean Age (SD) | 70.2 (15.4) | 68.2 (14.0) | 67.2 (16.2) | 68.8 (14.6) | 68.8 (13.3) |  |  |
| Median Age  (Q1, Q3) | 73.0 (61.0, 81.0) | 68.0 (59.0, 79.0) | 70.5 (57.0, 79.0) | 71.0 (59.0, 80.0) | 70.0 (59.0, 78.0) |  |  |
| <75 years, n (%) | 157 (52.5) | 166 (62.6) | 190 (60.9) | 160 (58.6) | 135 (60.5) | 1.03 (0.86, 1.24) | 0.726 |
| ≥75 years, n (%) | 142 (47.5) | 99 (37.4) | 122 (39.1) | 113 (41.4) | 88 (39.5) | 0.95 (0.75, 1.19) | 0.675 |
| Male, n (%) | 186 (62.2) | 156 (58.9) | 187 (59.9) | 175 (64.1) | 140 (62.8) | 1.02 (0.85, 1.22) | 0.793 |
| Female, n (%) | 113 (37.8) | 109 (41.1) | 125 (40.1) | 98 (35.9) | 83 (37.2) | 0.96 (0.76, 1.21) | 0.739 |
| MI+PE+AAD, n | 305 | 267 | 318 | 280 | 226 |  |  |
| Mean Age (SD) | 70.2 (15.3) | 68.2 (14.0) | 67.1 (16.2) | 68.7 (14.6) | 68.6 (13.4) |  |  |
| Median Age  (Q1, Q3) | 73.0 (61.0, 81.0) | 68.0 (59.0, 79.0) | 70.0 (57.0, 79.0) | 71.0 (59.0, 80.0) | 70.0 (59.0, 78.0) |  |  |
| <75 years, n (%) | 161 (52.8) | 167 (62.5) | 195 (61.3) | 166 (59.3) | 138 (61.1) | 1.04 (0.86, 1.24) | 0.698 |
| ≥75 years, n (%) | 144 (47.2) | 100 (37.5) | 123 (38.7) | 114 (40.7) | 88 (38.9) | 0.95 (0.75, 1.18) | 0.640 |
| Male, n (%) | 189 (62.0) | 157 (58.8) | 191 (60.1) | 181 (64.6) | 142 (62.8) | 1.02 (0.85, 1.22) | 0.797 |
| Female, n (%) | 116 (38.0) | 110 (41.2) | 127 (39.9) | 99 (35.4) | 84 (37.2) | 0.96 (0.76, 1.21) | 0.745 |

Poisson regression: 2020 (during RM) compared to previous years; RM, restrictive social measures.
