# Supplementary material for: Complications and mortality of cardiovascular emergency admissions during COVID-19 associated restrictive measures
Source: PLoS One. 2020 Sep 24;15(9):e0239801. doi: 10.1371/journal.pone.0239801 (PMC7514100; doi:10.1371/journal.pone.0239801)
Supplement: S2 Table — (DOCX) [file pone.0239801.s002.docx]

**S2 Table: Patient characteristics of admissions during (2020 during RM) and before (2020 before RM) COVID-19 associated RM.**

|  | 2020  (before RM) | 2020  (during RM) |
| --- | --- | --- |
| Sex, n | 272 | 226 |
| Male, n (%) | 170 (62.5%) | 142 (62.8%) |
| Female, n (%) | 102 (37.5%) | 84 (37.2%) |
| Age |  |  |
| Mean (SD) | 69.2 (14.4) | 68.6 (13.4) |
| Median (Q1, Q3) | 71.0 (58.0, 80.0) | 70.0 (59.0, 78.0) |
| <75, n (%) | 160 (58.8%) | 138 (61.1%) |
| ≥75, n (%) | 112 (41.2%) | 88 (38.9%) |
