# Supplementary material for: Complications and mortality of cardiovascular emergency admissions during COVID-19 associated restrictive measures
Source: PLoS One. 2020 Sep 24;15(9):e0239801. doi: 10.1371/journal.pone.0239801 (PMC7514100; doi:10.1371/journal.pone.0239801)
Supplement: S3 Table — (DOCX) [file pone.0239801.s003.docx]

**S3 Table: Number and mortality of admissions during (2020 during RM) and before (2020 before RM) COVID-19 associated RM.**

|  | 2020  (before RM) | 2020  (during RM) | RR (95% CI) | P value |
| --- | --- | --- | --- | --- |
| MI, n | 170 | 148 | 0.87 (0.70, 1.08) | 0.218 |
| Deaths, n (%) | 15 (8.8) | 16 (10.8) | 1.23 (0.60, 2.50) | 0.572 |
| PE, n | 100 | 75 | 0.75 (0.55, 1.01) | 0.060 |
| Deaths, n (%) | 3 (3.0) | 5 (6.7) | 2.22 (0.55, 10.84) | 0.274 |
| AAD, n | 2 | 3 | 1.50 (0.25, 11.39) | 0.657 |
| Deaths, n (%) | 1 (50.0) | 1 (33.3) | 0.67 (0.03, 16.86) | 0.774 |
| MI+PE, n | 270 | 223 | 0.83 (0.69, 0.99) | 0.035 |
| Deaths, n (%) | 18 (6.7) | 21 (9.4) | 1.41 (0.75, 2.68) | 0.282 |
| MI+PE+AAD, n | 272 | 226 | 0.83 (0.70, 0.99) | 0.040 |
| Deaths, n (%) | 19 (7.0) | 22 (9.7) | 1.39 (0.75, 2.60) | 0.289 |

Poisson regression: 2020 (during RM) compared to 2020 (before RM). RM, restrictive social measures.
